# Antiphospholipid Syndrome in a Patient With IgA Nephropathy: A Rare and Challenging Clinical Overlap

**DOI:** 10.7759/cureus.87462

**Published:** 2025-07-07

**Authors:** Usamah Al-Anbagi, Hatem M Abusriwil, Azeez Palol, Mohamed Emsalem, Alaa Abdelhamid, Abdulqadir J Nashwan

**Affiliations:** 1 Internal Medicine, Hazm Mebaireek General Hospital/Hamad Medical Corporation, Doha, QAT; 2 Medical Education, Hazm Mebaireek General Hospital/Hamad Medical Corporation, Doha, QAT; 3 College of Medicine, Qatar University, Doha, QAT; 4 Nursing and Midwifery Research, Hazm Mebaireek General Hospital/Hamad Medical Corporation, Doha, QAT

**Keywords:** anticoagulation, antiphospholipid syndrome (aps), case report, deep venous thrombosis (dvt), iga nephropathy (igan), pulmonary embolism (pe), thrombectomy

## Abstract

Antiphospholipid syndrome (APS) is a rare autoimmune condition associated with a heightened risk of blood clots in arteries and veins due to the presence of specific antibodies. IgA nephropathy (IgAN), a kidney disorder involving immune complex deposition, is another immune-mediated disease. The coexistence of APS and IgAN is highly uncommon. We describe a young adult male with biopsy-confirmed kidney involvement who developed a significant clot in the lower limb. Despite appropriate treatment, the clot progressed, leading to complications in the lungs. Further investigation revealed laboratory findings consistent with APS. The patient underwent advanced interventions including clot removal and vascular procedures, followed by long-term treatment to prevent further clotting. This study emphasizes the clinical complexity when these two immune-mediated conditions overlap and underscores the importance of early detection and collaborative care in managing such rare and serious presentations.

## Introduction

Antiphospholipid syndrome (APS) is a systemic autoimmune disorder characterized by recurrent arterial or venous thrombosis with persistently elevated antiphospholipid antibodies (aPL). Diagnosis requires positivity for at least one of three laboratory markers as follows: lupus anticoagulant, anticardiolipin antibodies, or anti-β2 glycoprotein I antibodies [1]. These antibodies promote thrombosis through immune-mediated mechanisms involving endothelial, platelet, and complement activation [2]. APS may present as a primary disorder or be associated with other autoimmune diseases, most commonly systemic lupus erythematosus [3]. Renal involvement in APS is termed antiphospholipid nephropathy, and it can range from thrombotic microangiopathy to large vessel thrombosis.

IgA nephropathy (IgAN) is the most common primary glomerulonephritis worldwide and is characterized by mesangial deposition of galactose-deficient IgA1-containing immune complexes, leading to renal inflammation and injury. Its pathogenesis is described by the “four-hit hypothesis” as follows: (1) production of galactose-deficient IgA1, (2) formation of anti-glycan autoantibodies, (3) immune complex deposition in the mesangium, and (4) resultant glomerular damage [4,5]. Diagnosis relies on kidney biopsy with immunofluorescence, and the Oxford classification system provides a standardized histopathological framework to assess disease severity and guide prognosis [6].

The coexistence of APS and IgA nephropathy is rare, and the overlapping immune mechanisms and vascular complications can present significant diagnostic and therapeutic challenges. We report a case of a 27-year-old male with a known history of biopsy-confirmed IgA nephropathy who presented with extensive left lower limb deep vein thrombosis and was ultimately diagnosed with antiphospholipid syndrome. This case underscores the rare coexistence of IgAN and APS, two distinct autoimmune conditions that seldom overlap, and highlights the aggressive thrombotic potential of APS. It also illustrates the complexity of diagnosing and managing extensive thromboembolic events in young patients with underlying renal autoimmunity and the importance of early multidisciplinary involvement and individualized anticoagulation strategies.

## Case presentation

Medical history

A 26-year-old male, a known case of IgAN, was diagnosed approximately 10 months before the current presentation. He reported being treated with budesonide 9 mg once daily in his home country, but he did not have any documentation to confirm this. He was confirmed by renal biopsy, presented to the emergency department with a three-day history of left thigh pain and fever. He also reported a single episode of vomiting the day before presentation. He denied any history of trauma, surgery, fall, recent long-distance travel, or prolonged immobility. His past medical history includes acute calculous cholecystitis, managed conservatively, and known gallbladder stones. He is unmarried and reports no sexual activity. There was no history of skin rash, joint pain, stiffness, or hair loss. The remainder of the history was unremarkable.

Examination

On examination, the patient was conscious, alert, and oriented. He was febrile, with a maximum recorded temperature of 39.2°C; other vital signs included a heart rate of 99 beats per minute (bpm), a respiratory rate of 16 breaths per minute, a blood pressure of 120/70 mmHg, and an oxygen saturation of 98% on room air. There was no pallor, cyanosis, clubbing, icterus, pedal edema, or lymphadenopathy. Local examination of the left thigh and inguinal region revealed mild tenderness and warmth over the left inguinal area, without noticeable swelling. Cardiovascular examination revealed normal heart sounds (S1 and S2) with no murmurs. Respiratory examination showed equal bilateral air entry with no added sounds. The abdomen was soft, lax, non-tender, and without hepatosplenomegaly. Neurologically, there were no focal deficits or signs of meningeal irritation. Skin examination revealed no rashes, nail changes, or oral/genital ulcers. There was no tenderness or swelling in the small joints of the hands, and examination of the wrists, elbows, shoulders, hips, knees, ankles, and feet was unremarkable.

Management and outcome

**Figure 1:**
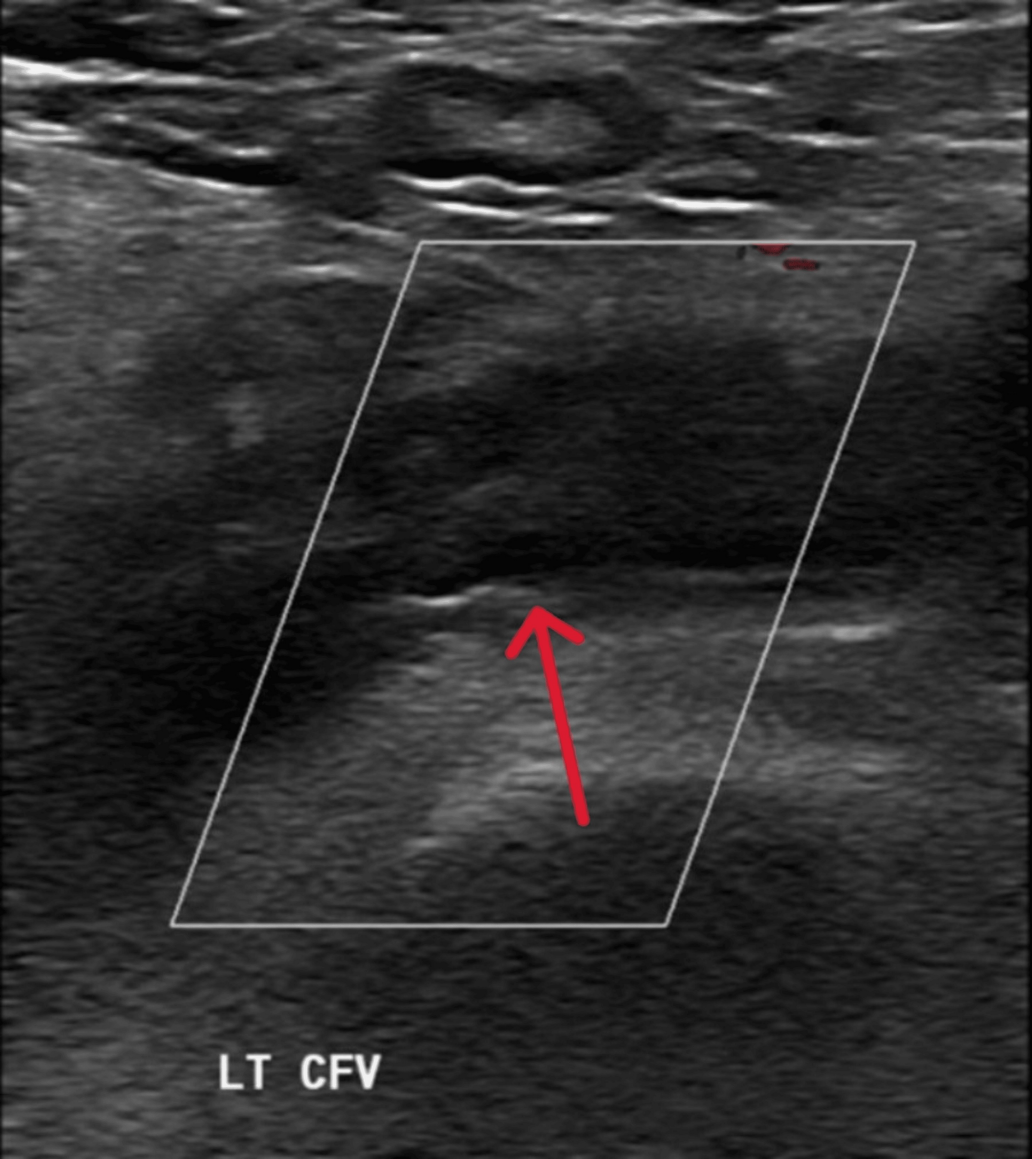
Color Doppler ultrasound showing deep vein thrombosis in the left common femoral vein. Color Doppler ultrasound image demonstrating thrombosis (arrow) in the left common femoral vein, consistent with deep vein thrombosis (DVT). The absence of flow within the vein and intraluminal echogenic material are characteristic findings. LT CFV: left common femoral vein

The initial routine investigations revealed mild leukocytosis, anemia, and a slightly low hematocrit (Table 1). A Doppler ultrasound of the left thigh, performed in the emergency department, confirmed extensive deep vein thrombosis (DVT) involving the left external iliac vein, left common femoral vein, and the great saphenous vein, with a patent inferior vena cava (Figure 1).

**Table 1:** Comprehensive laboratory parameters of the patient on admission, day three, and at discharge. ANA: antinuclear antibodies; anti-dsDNA: anti-double stranded DNA; anti-Ro52: anti-Ro52 antibody; anti-SS-A: anti-Sjögren’s-syndrome-related antigen A; anti-SS-B: anti-Sjögren’s-syndrome-related antigen B; anti-nucleosomes: anti-nucleosome antibodies; anti-Sm: anti-Smith antibodies; anti-RNP: anti-ribonucleoprotein antibodies; anti-histones: anti-histone antibodies; anti PCNA: anti-proliferating cell nuclear antigen antibodies; anti-ribosomal P protein: anti-ribosomal P protein antibodies; anti-Jo1: anti-histidyl-tRNA synthetase antibody; anti-AMA-M2: anti-mitochondrial M2 antibody; anti-centromere B: anti-centromere protein B antibody; anti-PM-Scl: anti-polymyositis-scleroderma antibody; β₂-glycoprotein IgM antibody: beta-2 glycoprotein I antibody (IgM); IgG: immunoglobulin G; IgM: immunoglobulin M; INR: international normalized ratio; AST: aspartate aminotransferase; ALT: alanine aminotransferase; TSH: thyroid stimulating hormone; FT4: free thyroxine, C3: complement component 3; C4: complement component 4; PT: prothrombin time; aPTT: activated partial thromboplastin time

Parameters	1st day	3rd day	On discharge	Reference values
Anti-cardiolipin IgG antibody (U/mL)	29	-	-	<15
Anti-cardiolipin IgM antibody (U/mL)	22	-	-	<10
ANA profile (includes anti-dsDNA, anti-Ro52, anti-SS-A, anti-nucleosomes, anti-Sm, anti-RNP, anti-histones, anti-PCNA, anti-SS-B, anti-ribosomal P protein, anti-Jo1, anti-AMA-M2, anti-centromere B, anti-PM-Scl antibodies, β₂-glycoprotein IgM antibody)	All were negative	-	-	Negative
Factor XII (%)	74	-	-	70-150
Factor V	Normal	-	-	Normal
Lupus screen (s)	145	-	-	30.4-45.3
Lupus anti-coagulant	Positive	-	-	Not detected
Protein C activity (%)	68	-	-	70-140
Protein S activity (%)	91	-	-	72-126
C 3 (g/L)	1.71	-	-	0.9-1.8
C 4 (g/L)	0.21	-	-	0.1-0.4
Anti-thrombin activity (%)	97	-	-	79.4-130
INR	1.8	1.5	2	<1.1
Prothrombin time (s)	20	17	22.3	9.5-12.5
Activated partial thromboplastin time (s)	75	96	105.7	25-36.5
Total leukocytes (×10^3^/µL)	11.1	7.8	6.7	6.2
Serum hemoglobin (g/dL)	11.3	9.9	10.1	13-17
Hematocrit (%)	34.6	31.1	30.5	40-50
Serum potassium (mmol/L)	4.1	4.4	4.5	3.5-5.3
Serum sodium (mmol/L)	133	141	134	133-146
Serum magnesium (mmol/L)	0.86	0.84	-	0.7-1
Serum urea (mmol/L)	3.4	3.1	4.1	2.5-7.8
Serum creatinine (µmol/L)	92	78	87	62-106
Serum glucose (mmol/L)	5.6	5.9	6.4	<11.1
Serum albumin (g/L)	37	27	32	35-50
Serum total protein (g/L)	78	70	78	60-80
AST (IU/L)	20	28	21	0-41
ALT (IU/L)	30	34	37	0-41
Alkaline phosphatase (U/L)	51	56	53	40-129
TSH (mIU/L)	3.6	-	-	0.3-4.2
FT4 (pmol/L)	18.7	-	-	11-23.3
Serum total bilirubin (mg/dL)	26	10	9	0-21
Homocysteine level (mmol/L)	7	-	-	0-12
Urine total protein (g/24 h)	0.91	-	-	0.03-0.15

The patient was admitted with extensive deep vein thrombosis (DVT) and a background of IgAN and started on therapeutic enoxaparin (1 mg/kg twice daily). He did not present with features of nephrotic syndrome. Urine dipstick showed only trace proteinuria, and the 24-hour urine protein was 0.9 g. A comprehensive thrombophilia workup was also performed, which revealed positive anticardiolipin IgG and IgM antibodies, a positive lupus anticoagulant, and a prolonged activated partial thromboplastin time (APTT) that failed to correct on a 1:1 mixing study, suggestive of the presence of an inhibitor (Table 1).

**Figure 2:**
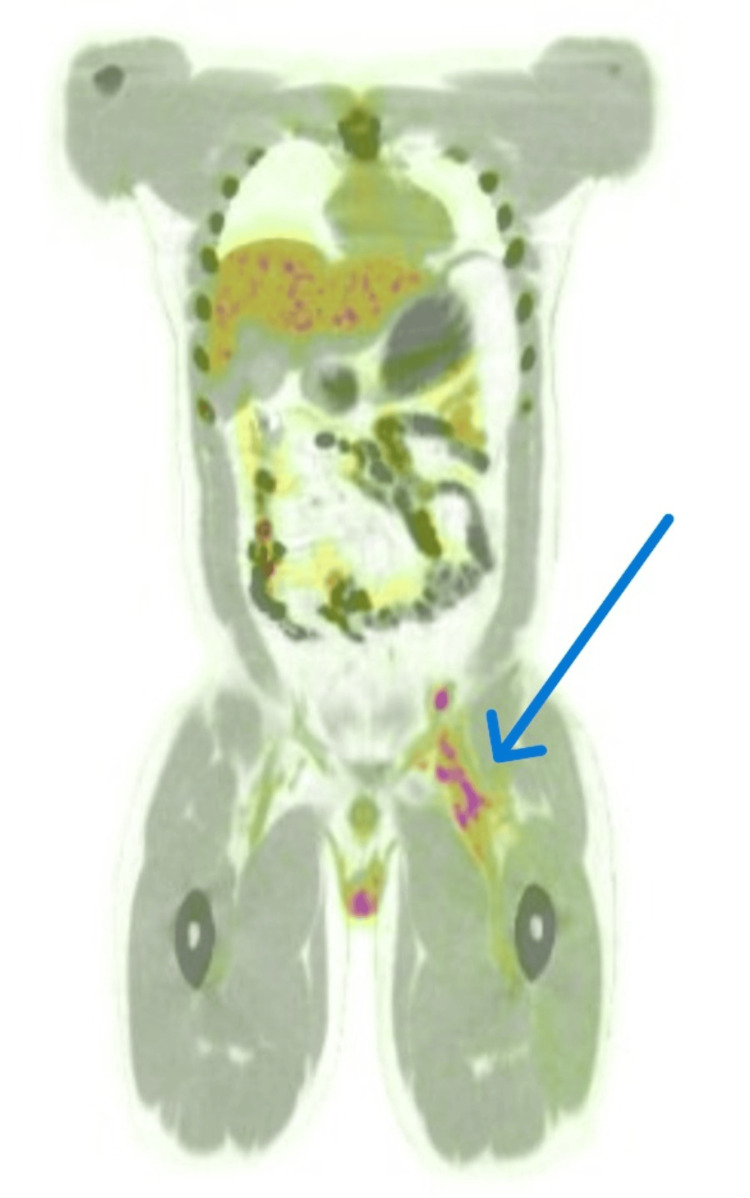
PET scan indicating thrombophlebitis of the left external iliac and femoral veins. Positron emission tomography (PET) scan image demonstrating increased radiotracer uptake along the dilated left external iliac and femoral veins (arrow), consistent with thrombophlebitis secondary to venous thrombosis. The localized uptake reflects the inflammatory response associated with the thrombotic process.

These findings confirmed the diagnosis of APS. Hematology, nephrology, and rheumatology teams were involved. The patient continued on therapeutic enoxaparin and was also started on empirical ceftriaxone, which was later de-escalated to oral amoxicillin/clavulanate 1 g twice daily after a negative septic workup. A PET scan was performed to rule out vasculitis or malignancy. It showed only thrombophlebitis related to known venous thrombosis in the left external iliac and femoral veins, with reactive lymph nodal uptake and no evidence of malignancy or vasculitis (Figure 2). The patient was then started on warfarin 10 mg with enoxaparin overlap until the target INR of 2-3 was achieved, and he was discharged on warfarin 6 mg daily with a follow-up appointment scheduled in two weeks.

**Figure 3:**
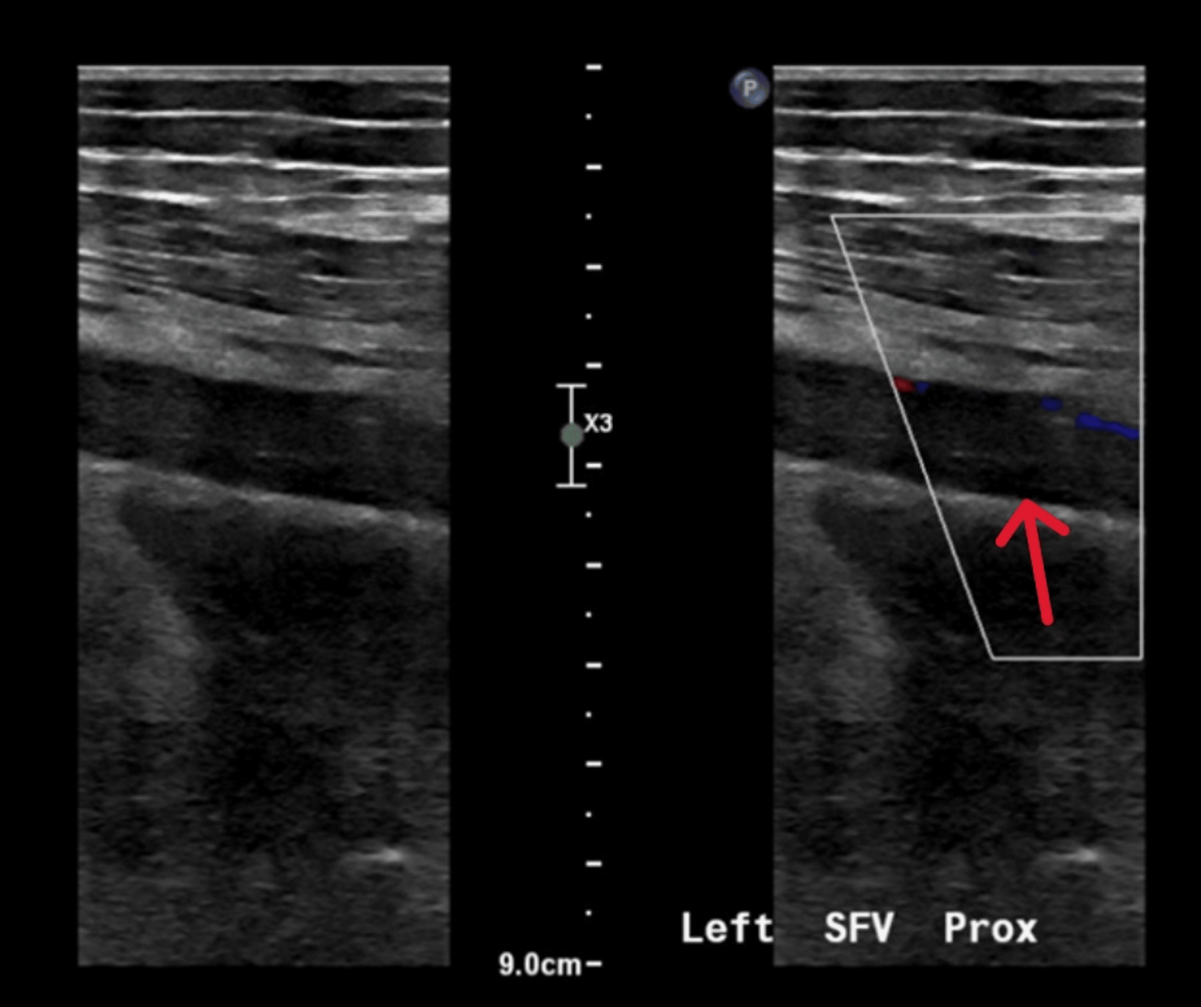
Doppler ultrasound revealing deep vein thrombosis of the left superficial femoral vein. Doppler ultrasound image showing deep vein thrombosis (DVT) of the left superficial femoral vein (arrow), characterized by intraluminal echogenic material and disturbed blood flow. The abnormal Doppler signal pattern indicates impaired venous return due to thrombosis. SFV: superficial femoral vein

However, five days later, he was readmitted with fever and worsening left thigh pain. Repeat Doppler ultrasound showed extension of the thrombus to include the popliteal, superficial femoral, common femoral, and iliac veins (Figure 3).

**Figure 4:**
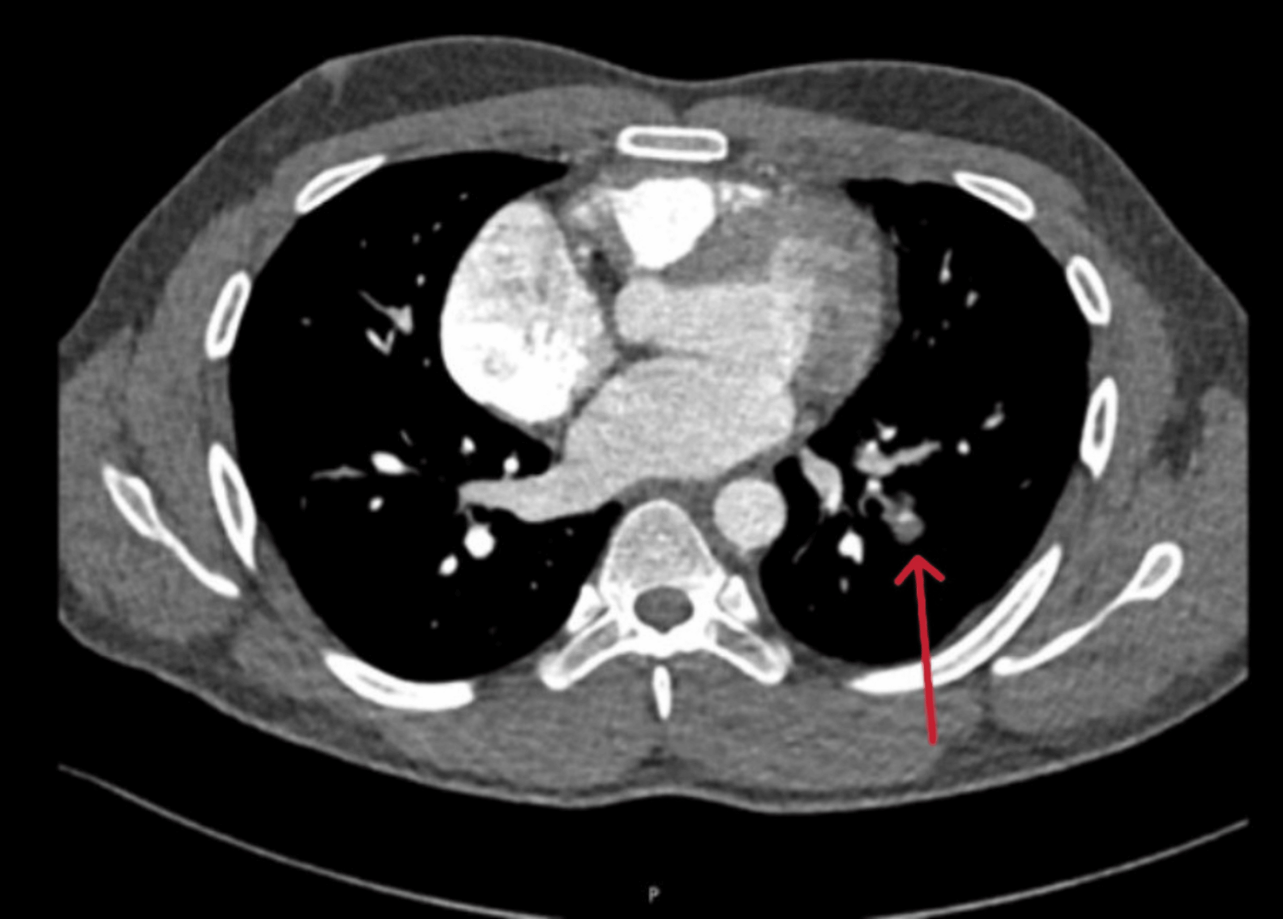
Computed tomography pulmonary angiography revealing pulmonary embolism. Axial view of a computed tomography (CT) pulmonary angiography image demonstrating a filling defect within the pulmonary artery (arrow), indicative of acute pulmonary embolism. The defect represents an intravascular thrombus obstructing pulmonary blood flow.

His INR was above the target range at 4.5, so warfarin was temporarily withheld. The hematology, rheumatology, vascular surgery, and interventional radiology teams were consulted for possible thrombectomy and inferior vena cava (IVC) filter placement. Orthopedics also evaluated the patient and ruled out coth clinically and radiologically. Compartment syndrome CT venogram revealed extensive left-sided DVT with caliber reduction of the external iliac vein and an extensive filling defect involving the superficial femoral, common femoral, and proximal external iliac veins. A few days later, the patient underwent left lower limb thrombolysis, mechanical thrombectomy, venoplasty, and stenting. Three days later, he developed left-sided chest pain, and CT pulmonary angiography revealed bilateral segmental pulmonary emboli with left lower lobe pulmonary infarction (Figure 4).

As a result, the target INR was increased to 2.5-3.5, which was achieved with warfarin 6 mg. The patient was discharged in stable clinical condition with an urgent follow-up appointment to ensure close monitoring.

## Discussion

This case highlights a rare and clinically significant overlap between APS and IgAN. The patient, previously diagnosed with IgAN, presented with extensive DVT and was subsequently diagnosed with APS, confirmed by the presence of lupus anticoagulant, anticardiolipin antibodies, and an uncorrected prolonged activated partial thromboplastin time. Despite being on therapeutic anticoagulation, he developed bilateral pulmonary embolism, indicating ongoing thrombotic activity. This case underscores the diagnostic challenge of identifying APS in patients with underlying autoimmune kidney disease and emphasizes the heightened thrombotic risk and management complexity when these two conditions coexist.

APS is a systemic autoimmune condition primarily characterized by venous or arterial thrombosis and adverse pregnancy outcomes, such as recurrent miscarriages or fetal loss [1]. Other clinical features may include thrombocytopenia, livedo reticularis, and valvular heart disease. The diagnosis relies on both clinical findings and persistent laboratory evidence of moderate to high levels of aPL, which include lupus anticoagulant (LA), anticardiolipin (aCL) antibodies, and anti-beta2 glycoprotein I (anti-β2GPI) antibodies [7]. APS can be classified as primary or secondary, with the latter being most commonly associated with systemic lupus erythematosus (SLE). Subtypes include thrombotic APS, obstetric APS, microvascular APS, and catastrophic APS (CAPS), a rare form involving multiorgan failure [8]. The 2023 ACR/EULAR classification criteria offer more stringent definitions for research purposes, but clinical judgment remains essential for diagnosis and management [8].

IgAN is an autoimmune glomerular disease driven by abnormal mucosal IgA responses, characterized by the overproduction of galactose-deficient IgA1 (Gd-IgA1) molecules that trigger the formation of specific IgG and IgA autoantibodies [9]. These antibodies form circulating immune complexes that deposit in the glomerular mesangium, leading to inflammation and kidney injury through complement activation and mesangial proliferation. The “four-hit” hypothesis outlines this pathogenic cascade, in which mucosal infections, dysregulated responses to antigens, and alterations in the gut microbiome can exacerbate the condition. IgAN is the most common primary glomerulonephritis worldwide, usually affecting young adults, with male predominance in Western populations [10]. It typically presents as episodes of gross hematuria associated with infections or as persistent microscopic hematuria with proteinuria and hypertension [11]. Diagnosis requires a kidney biopsy, often performed when proteinuria is significant or renal function declines. Treatment aimed to prevent progression to end-stage kidney disease through blood pressure control, reduction of proteinuria, and management of cardiovascular risk, with immunosuppressive therapy reserved for high-risk patients [12,13].

The simultaneous occurrence of APS and IgAN is infrequent, with only a few cases documented in the literature. APS is predominantly associated with systemic SLE, and its manifestation in patients without SLE, termed primary APS, is less common. Renal involvement in APS typically presents as thrombotic microangiopathy or large-vessel thrombosis, whereas IgAN is characterized by mesangial deposition of IgA-containing immune complexes. The coexistence of these two distinct pathologies in a single patient is unusual. A case report by Baradaran and Nasri detailed a patient with cutaneous vasculitis, IgAN, and APS, further emphasizing the rarity of this triad [14].

Additionally, a study by Nzerue et al. discussed the incidental finding of lupus anticoagulant in a patient with IgAN, suggesting a possible but unconfirmed association [15]. These cases underscore the importance of considering APS in patients with IgAN who present with atypical symptoms or thrombotic events. Despite the limited number of reported instances, IgAN presents significant prognostic challenges, even in the absence of APS. Renal involvement in APS, particularly in the form of thrombotic microangiopathy (TMA), is associated with a high risk of progression to end-stage renal disease (ESRD) and increased mortality. A study by Tektonidou highlighted that APS nephropathy (APSN) lesions, such as TMA, fibrous intimal hyperplasia, and glomerular sclerosis, are prevalent in patients with APS and correlate with poor renal outcomes. The presence of APSN in IgAN patients complicates the clinical picture, as both conditions contribute to renal damage. This overlap necessitates vigilant monitoring and a multifaceted therapeutic approach to mitigate renal deterioration [16].

However, managing patients with coexisting APS and IgAN remains challenging and is not well-defined in the literature. The coexistence of a thrombotic disorder and an immune-mediated glomerulonephritis poses unique therapeutic considerations. Immunosuppressive agents for IgAN, such as corticosteroids, must be carefully balanced against the risk of bleeding associated with anticoagulation, particularly in patients with impaired renal function. There is a lack of randomized controlled trials addressing this overlap, and decisions are often made on an individual basis. Future studies should aim to clarify the clinical course and optimal management of patients with this dual pathology. Clinicians should maintain a high index of suspicion for APS in patients with IgAN who present with thrombotic events, as early recognition and tailored treatment are crucial for improving both renal and systemic outcomes.

## Conclusions

This case underscores a rare but clinically significant coexistence of antiphospholipid syndrome and IgA nephropathy, revealing a complex interplay between autoimmune thrombophilia and glomerular injury. The patient’s rapid thrombotic progression despite appropriate anticoagulation highlights the aggressive nature of APS when superimposed on existing renal pathology. This dual diagnosis demands heightened clinical vigilance, multidisciplinary coordination, and individualized management strategies. As literature on this overlap remains scarce, this case adds valuable insight and reinforces the critical need for further research to guide evidence-based care in such challenging scenarios.

## References

[1] Miyakis S, Lockshin MD, Atsumi T (2006). International consensus statement on an update of the classification criteria for definite antiphospholipid syndrome (APS). J Thromb Haemost.

[2] Garcia D, Erkan D (2018). Diagnosis and management of the antiphospholipid syndrome. N Engl J Med.

[3] Ünlü O, Zuily S, Erkan D (2016). The clinical significance of antiphospholipid antibodies in systemic lupus erythematosus. Eur J Rheumatol.

[4] Barratt J, Feehally J, Smith AC (2004). Pathogenesis of IgA nephropathy. Semin Nephrol.

[5] Dotz V, Visconti A, Lomax-Browne HJ (2021). O- and N-glycosylation of serum immunoglobulin A is associated with IgA nephropathy and glomerular function. J Am Soc Nephrol.

[6] Coppo R, Troyanov S, Camilla R (2010). The Oxford IgA nephropathy clinicopathological classification is valid for children as well as adults. Kidney Int.

[7] Devreese KM, Bertolaccini ML, Branch DW (2025). An update on laboratory detection and interpretation of antiphospholipid antibodies for diagnosis of antiphospholipid syndrome: guidance from the ISTH-SSC Subcommittee on Lupus Anticoagulant/Antiphospholipid Antibodies. J Thromb Haemost.

[8] Barbhaiya M, Zuily S, Naden R (2023). The 2023 ACR/EULAR Antiphospholipid Syndrome Classification Criteria. Arthritis Rheumatol.

[9] Hiki Y, Odani H, Takahashi M (2001). Mass spectrometry proves under-O-glycosylation of glomerular IgA1 in IgA nephropathy. Kidney Int.

[10] Galla JH (1995). IgA nephropathy. Kidney Int.

[11] Donadio JV, Grande JP (2002). IgA nephropathy. N Engl J Med.

[12] Pitcher D, Braddon F, Hendry B (2023). Long-term outcomes in IgA nephropathy. Clin J Am Soc Nephrol.

[13] Coppo R, Troyanov S, Bellur S (2014). Validation of the Oxford classification of IgA nephropathy in cohorts with different presentations and treatments. Kidney Int.

[14] Baradaran A, Nasri H (2013). Rare association of cutaneous vasculitis, IgA nephropathy and antiphospholipid antibody syndrome with tuberculous lymphadenitis. Clinics (Sao Paulo).

[15] Nzerue CM, Hewan-Lowe K, Pierangeli S, Harris EN (2002). "Black swan in the kidney": renal involvement in the antiphospholipid antibody syndrome. Kidney Int.

[16] Tektonidou MG (2018). Antiphospholipid syndrome nephropathy: from pathogenesis to treatment. Front Immunol.

